# The social environment of talent development in youth sport

**DOI:** 10.3389/fspor.2023.1127151

**Published:** 2023-02-13

**Authors:** Kristoffer Henriksen, Natalia Stambulova

**Affiliations:** ^1^ Institute of Sport Science and Clinical Biomechanics, University of Southern Denmark, Odense, Denmark; ^2^ School of Health and Welfare, Halmstad University, Halmstad, Sweden

**Keywords:** ecological psycholgy, youth sport, athletic talent, sport environment, holistic ecological approach, athletic talent development environment

## Abstract

During the last decade, talent identification and development research that favours an individual perspective has been complemented by a focus on young athletes' social environments, termed “athletic talent development environments” (ATDEs). Two major lines of research have created a foundation for an ecological vision of talent development as the mutual accommodation between athletes and their ATDEs and of career development as an athlete's journey through various athletic and non-athletic environments. The Talent Development Environment Questionnaire allows the quantitative screening of athletes' environments, while the holistic ecological approach (HEA) promotes in-depth qualitative case studies of ATDEs. In this chapter, we focus mainly on the HEA, including: (a) two models that combine to illustrate an ATDE; (b) a summary of empirical case studies of successful environments in various sports and countries, culminating in a set of shared features of ATDEs that promote athletes' wellbeing and athletic and personal development; (c) an overview of recent trends within HEA (e.g. interorganisational collaboration in talent development) and (d) recommendations for coaches and sport psychology consultants, emphasising the importance of integrating efforts across the whole environment and building strong and coherent organisational cultures. In the discussion, we elaborate on developing the HEA discourse and point to future challenges for researchers and practitioners.

## Introduction

In August 2021, a Danish sailor won the Olympic gold medal in her event in Tokyo. While she was on the water, her Danish teammates watched the event together, their eyes filled with tears of joy and pride as she crossed the finish line. They felt they had a big share in that victory, and in the post-race interview the winner was quick to give them credit and highlight their important role in her success. That same year, she turned 30, neared the end of her university degree and was offered a way into the world of professional sailing. The media naturally took an interest in her plans and specifically her potential for a repeat Olympic performance. After half a year of silence, she announced that she had decided to aim for the Olympics again. Explaining her motivation, she did not say that another Olympic medal would change her life or that she wanted to taste the sweetness of success and nationwide recognition once again. Instead, she highlighted her training environment in which she could grow, learn, give back and feel at home, saying that with this team her journey towards the next Games would not only be fun but also realistic.

No one makes it on their own. Borrowing from an old African adage, it takes a village to raise an athlete, and when reflecting on their talent development pathways, elite athletes acknowledge people without whom they never would have made it. Elite athletes' tales often illustrate that successful talent development is a journey through good environments that have supported their striving as well as their thriving.

15–20 years ago, talent development research was dominated by individual perspectives (1), whereby researchers aimed to discover the unique characteristics (e.g., 2, 3) and pathways (e.g., 4, 5) of elite athletes to inform talent identification and development initiatives. Inspired by ecological perspectives in sport-related learning and decision making (see special issue 6);, two research groups in parallel initiated investigation of the role of the environment in talent development in sports. In Scotland, Martindale and colleagues (7) developed a survey that could assess the quality of an athletic talent development environment (ATDE), and in Denmark, Henriksen and colleagues completed a series of innovative in-depth case studies of successful ATDEs in Scandinavia (8, 9, 10). Today, the ecology of talent development discourse has matured, as visible in two recent reviews summarising key findings of more than a decade of ecological talent development research and related practice (11, 12).

### The ecology of talent development in sport

In this chapter, we discuss research regarding ATDEs in youth sport and practical implications – both grounded in *the holistic ecological approach* (HEA). By *ecological*, we mean the focus on the athletes' environment that affects their development; *holistic* refers to a view of the environment as a complex and dynamic whole that consists of multiple interrelated settings, levels and domains (13, 14).

We begin with the model of Effective Talent Identification and Development Procedures by Martindale and colleagues (15) that was developed based on interviews with experienced coaches about successful ATDEs' contributions to the development of young athletes. The model formed the basis of the Talent Development Environment Questionnaire, TDEQ (7), that measures five features of an environment that fosters talent development: (a) long-term aims and methods; (b) wide-ranging and coherent messages and support; (c) emphasis on talent development rather than early selection; (d) individualised and ongoing development; and (e) an integrated and holistic system. The TDEQ and a subsequent revised version, TDEQ5 (16), have been used to gauge strengths and weaknesses of specific ATDEs to assist efforts to improve environments (e.g., 17). The instrument was further used to investigate associations between features of ATDEs and athletes' development. Although the various modifications of the structure warrant caution, research has demonstrated that athletes' favourable perception of their ATDE was linked positively to the satisfaction of their basic needs, mental toughness and wellbeing (18, 19) and negatively associated with burnout (20, 21).

**Figure 1:**
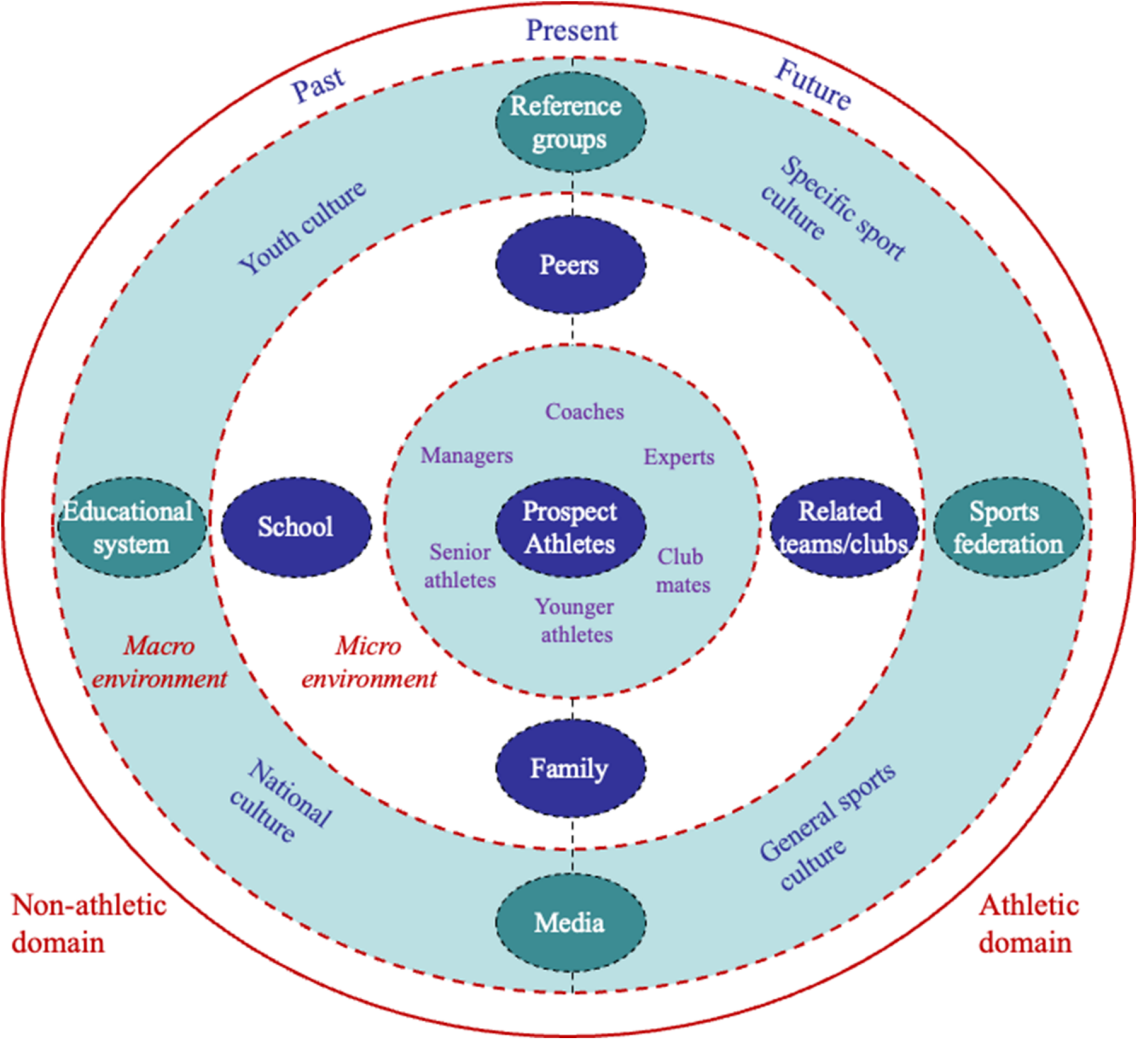
The athletic talent development environment (ATDE) working model. Reprinted with permission.

We now move to *the holistic ecological approach* (HEA) that offers a case study (qualitative) approach to investigating the structure, culture and inner workings of ATDEs that have had varying degrees of success in helping athletes to make the junior-to-senior transition (14). To aid case studies, two working models (8) were created by taking inspiration from ecological psychology, systems theory and cultural psychology (22–24). Figure 1 presents the ATDE working model as a framework for describing the roles and functions of the different components and relations within an environment. The prospective young elite athletes appear at the centre of the model, and other ATDE's components are structured into two levels (micro and macro) and two domains (athletic and non-athletic). The micro level refers to the environment in which the prospective elite athletes spend a good deal of their daily lives. The macro level refers to social settings, which affect but do not contain the athletes, as well as to the values and customs of the cultures to which the athletes belong. The athletic domain covers the part of the athletes' environment that is directly related to sport, whereas the non-athletic domain presents all the other spheres of the athletes' lives. The outer layer of the model represents the past, present and future of the ATDE, emphasising that the environment is dynamic.

**Figure 2:**
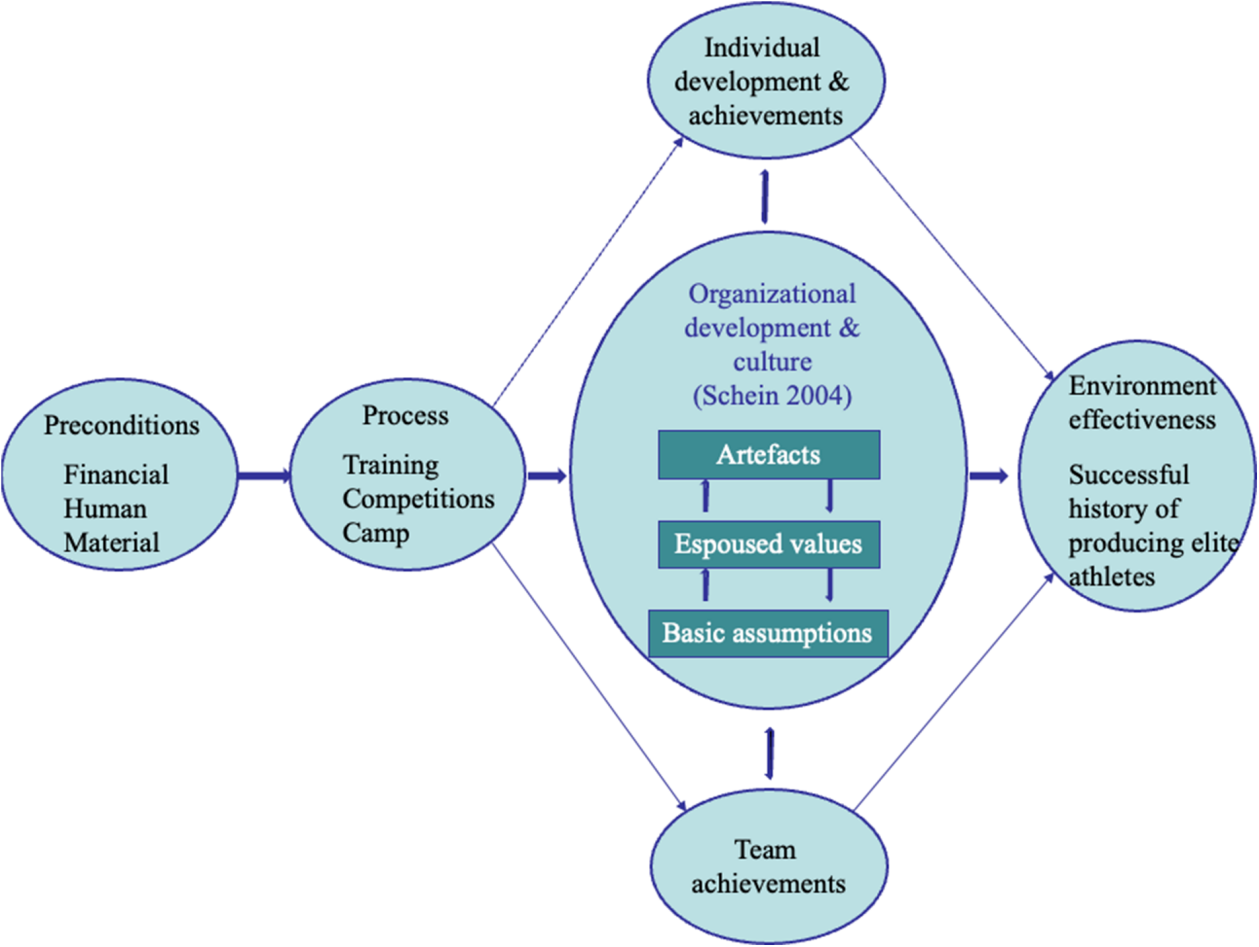
The environment success factors (ESF) working model. Reprinted with permission.

The Environment Success Factors (ESF) working model (Figure 2) predicts that the ATDE's success is a result of the interplay between *preconditions, processes, individual* and *team development and achievements*, with *organisational culture* serving to integrate these elements.

The model's starting point is the environment's *preconditions* (e.g., human, material and financial), all of which are necessary but not sufficient for success. The model then illustrates how the daily *processes* (e.g., training, camps and competitions) lead to three outcomes: athletes' individual development and achievements (e.g., psychosocial and athletic skills), team achievements and organisational development and culture. *Organisational culture* (25) is central to the ESF model and consists of: cultural artefacts (e.g., stories, customs and physical manifestations such as clothing and organisation charts), espoused values (i.e., principles, goals and standards that the organisation shows to the world) and basic assumptions (i.e., taken for granted and serving as underlying reasons for actions). Key basic assumptions are integrated into a *cultural paradigm*, guiding the socialisation of new members and providing stability. Organisational culture is seen as an integrative factor of the ADTE's effectiveness in helping talented young athletes to develop into senior elite athletes (14). Broadly speaking, successful ATDEs are environments that promote athletes' wellbeing and long-term athletic and personal development (12).

The HEA models have been tested and empirically validated through in-depth and real-time case studies of successful ATDEs. Examples of these studies include (but are not limited to) the Danish national 49er sailing team (8), a Swedish track and field club (9), a Norwegian kayak team situated in an elite sport high school (10), soccer academies across Europe (26–29), two handball clubs in Denmark and Norway (30) and a world-class trampoline environment in Canada (31). In contrast, one study was focused on a less successful golf environment in Denmark (32). Further, cross-case comparisons (33) allowed us to conclude that even though all environments are unique (no two environments are ever the same), successful ATDEs employ many of the same principles in their work. These principles were implemented in different ways, and some environments compensated for a weakness in one feature by a strong presence of another. Thus, the uniqueness of each environment was expressed in how they implemented the common principles. In Table 1, we summarise the shared features of ATDEs, providing descriptions of their *positive* (research based) and *opposite* poles (inferred logically or grounded in the study of the less successful environment). The shared features fall within two overall categories related to the structure and the culture of the environment.

**Table 1:** Shared features of successful ATDE*s.*

Features of successful ATDEs	Descriptors	Opposite Poles
**Structure**		
Integration of efforts	Coordination and communication between sport, school, family and other components; athletes experience synergy	Lack of communication; conflicting interests; athletes experience contradicting demands
Training groups with supportive relationships	Opportunities for inclusion in a training community with supportive relationships and friendships	Individualized training programs at an early stage; training alone; rivalry and low cohesion
Proximal role models	Opportunities to train with the elite athletes who are willing to pass on their knowledge	Boundaries between athletes at different levels. Elite level athletes keep their secrets
Support of sporting goals by the wider environment	School, family and friends acknowledge the athletes’ dedication to sport	Non-sport environment shows lack of understanding of elite sport
**Organizational culture**		
Coherent organizational culture	Coherence between espoused values and actions provides stability	Fragmented culture; espoused values do not correspond to actions; uncertainty
Support for the development of psychosocial skills	Opportunities to develop competences that are of benefit outside sport; considering athletes as “whole human beings”	Focus solely on sport; excessive control from coaches; focus on relative performance before personal improvement
Training that allows for diversification	Opportunities to sample different sports during early phases; focus on versatile basic sport skills in training	Promoting early specialization and sport specific skills only; considering other sports as rivals
A room for free initiative	Opportunities to organize training at own initiative across age- and training groups.	Inaccessible facilities and high training loads demotivate athletes to train outside formal training.
Knowledge sharing	Coaches share knowledge inside the ATDE and with coaches in other ATDEs	Coaches protect their “secrets” and consider other coaches only as rivals.
Focus on long-term development	Focus on long-term development of the athletes; age-appropriate training	Focus on early success; kids train like miniature elite athletes.

To continue with shared features, a recent scoping review of ATDEs (12) covered 44 studies published mainly during the last decade. In the analysis of the studies, the authors focused on *positive* (wellbeing, long-term athletic and personal development) and *less positive* (illbeing, limited athletic and personal development) *talent development outcomes* and related *functional* and *dysfunctional features of ATDEs*. Such an explicitly holistic definition of environment success is a welcome addition to the original literature that defined success as a track record of developing elite athletes but found that successful environment did in fact promote holistic development (11). The features were further sorted into four categories with clear connections to the ESF model (see Figure 2 and Table 1): preconditions, organisational culture, integration of efforts and quality holistic preparation. To provide a glimpse into the authors' preliminary conceptual framework, on the functional side, *preconditions* include skilled staff, accessible role models and system-wide support; *organisational culture* is characterised by an empowering climate, psychological safety and coherent and lived values; *integration of efforts* includes social relationships outside of sport and collaboration among stakeholders; and *quality holistic preparation* focuses on holistic personal development and long-term athletic preparation. On the dysfunctional side, examples of corresponding features refer to limited and unskilled staff, lack of role models and facilities, promoting winning at all costs, isolation and lack of stakeholder collaboration, lack of interest in the athletes as persons and inhibited preparation. The authors conclude that ATDEs weighted in favour of the functional features (and compensating for or eliminating dysfunctional ones) will provide positive outcomes in regard to athletes' wellbeing and athletic and personal development.

A holistic and ecological outlook has clear implications for practitioners. Coaches and talent development managers are encouraged to look beyond their training sessions and take an interest in providing a whole environment that is conducive for the athletes' development. Related to the structure of the environment, coaches might coordinate training camps and intense training periods with school exam periods, deliberately recruit and support role models, ensure communication within athletes' micro-environments (e.g., club, academy and national team training) and promote supportive training groups. In relation to organisational culture, coaches can acknowledge their role as cultural leaders (34), strive to develop a cohesive culture, stimulate athletes' free initiatives and maintain a long-term development focus. Sport psychology consultants are encouraged to conduct their interventions inside the athletes' natural settings and aim to optimise not only an athlete's individual psychological skills but the entire environment.

### New trends in the holistic ecological research and practice

As HEA has gained popularity, the approach has found its way into new but related domains of research. Zooming in on the ATDE's macro level, a case study in Danish swimming (35) and subsequent case studies in multiple sports (36) examined the successful collaboration in talent development management between a federation, a municipality and a local club, termed “an organisational triangle”. This research demonstrated that successful interorganisational collaboration in talent development required a shared philosophy and collaborative decisions, which allowed for coherent actions that would eventually lead to outcomes beneficial for the local athletes/clubs.

The next expansion of HEA has been its application in the study of Dual Career Development Environments (DCDEs) supporting athletes' efforts in combining their competitive careers with education or work. Seven case studies of successful DCDEs were conducted within the European Project “Ecology of Dual Careers” (37) based on adapted versions of the original ATDE and ESF models (38, 39). The further cross-case analysis led to the identification of ten essential features of DCDEs, such as a dedicated DC support team, integration of efforts, mentorship and access to expert support as characteristics of a holistic DCDE structure. Whole person and empowerment approaches, flexible solutions, care of mental health and a proactive approach to the development of the environment further described the shared dual career philosophy (40).

The most recent research project explored the nature of underserved athletic talent development environments (UATDE). An exploration of the career pathways of ten American professional athletes with low socioeconomic backgrounds highlighted the challenging circumstances they had to overcome to achieve athletic success and how their time in a UATDE had lasting ramifications in their lives (41). An interview study with stakeholders working in or with athletes from UATDEs unearthed specific challenges faced in UATDEs and demonstrated how developing within such environments impacted athletes even after they reached the college and professional levels of sports (42). Finally, a case study applied the HEA as a lens to examine a specific UATDE in basketball (43) with adapted versions of the original HEA models used to guide data collection. This research demonstrated that operation of the UATDE was significantly influenced by the underserved community in which it was embedded and that the team's roster comprised athletically talented but psychosocially vulnerable players, requiring the support team to expend considerable resources in supporting the psychosocial development of their players. Nevertheless, the UATDE managed to support the athletes in making a successful transition to a professional career and a better life because of a small but dedicated support team and a cultural paradigm that set the person before the performer and catered to the athletes' needs beyond the basketball court, and which was carefully maintained by the head coach as a cultural leader.

## Discussion: Major achievements and challenges for the future

The HEA research and practice were initially constructed in the overlap between talent development and career development discourses and have enriched both. Over a little more than a decade, we have observed how the HEA sport psychology discourse as a co-constructed and shared body of knowledge about athletes' environments (e.g., definitions, values and research-related and applied frameworks) has matured and created fruitful intersections with mental health, cultural and organisational sport psychology discourses. Combining the HEA with the holistic developmental approach (44) and a focus on athletes' mental health (45) led to a new understanding of career development as the pursuit of career excellence that sustains a healthy, successful and long-lasting career in sport and life (46). The HEA helps to understand that striving for career excellence is a dynamic process of mutual accommodation between athletes and their whole environments. Athletes use the environmental resources, just as they contribute to the success and development of their environments.

Being able to observe and contribute to development of the HEA discourse, we foresee the following lines of its further development:

• We envision successful athlete development as a journey through good environments that support the athletes' sport and personal development. This vison drives us to suggest that studies of successful and less successful environments at different career stages are needed, for example, youth sport and elite sport environments that come before and after the talent stage. For Bronfenbrenner (13), time was a key feature of the developmental processes, and research should pay more attention to the *journey*.
• Because of athletes' “travel” between different environments, career assistance programmes in the future should focus on helping athletes to prepare for, and cope with, environmental transitions as a supplement to the current focus on transitions between career stages.
• Environments can be resources and/or barriers for athletes' development and wellbeing. Recently, several elite athletes openly confessed their mental ill health, often pointing at abusive sport environments as key reasons. Keeping talented young athletes in sport requires the promotion of healthy and safe climates in ATDEs by strengthening their functional features while eliminating or compensating for dysfunctional ones.
• The important role of health and wellbeing as a resource for performance and personal development is not limited to athletes. Therefore, researchers and practitioners are encouraged to investigate and promote healthy environments for coaches, managers, peers, parents and sport psychology consultants, who influence the athletes.
• The HEA is expanding into new horizons (e.g. DCDE, UATDE), and we expect researchers to gradually give more nuanced and contextualised recommendations to developing good social environments for young athletes across sporting contexts.

No one makes it on their own. We invite researchers and practitioners worldwide to collectively contribute to the HEA-informed research and practice to create environments facilitating athletes' successful career excellence pursuits.

## Author contributions

The manuscript was developed on the initiative of KH. KH and NS both contributed to development of intellectual content, structure and key messages: KH wrote the majority of the text with NS concising and providing feedback. Both authors contributed to the article and approved the submitted version.
